# Publisher Correction: Approximation of nearly-periodic symplectic maps via structure-preserving neural networks

**DOI:** 10.1038/s41598-023-38690-w

**Published:** 2023-07-18

**Authors:** Valentin Duruisseaux, Joshua W. Burby, Qi Tang

**Affiliations:** 1 Department of Mathematics, University of California San Diego, La Jolla, CA 92093 USA; 2 Theoretical Division, Los Alamos National Laboratory, Los Alamos, NM 87545 USA

Correction to: *Scientific Reports* https://doi.org/10.1038/s41598-023-34862-w, published online 23 May 2023.

The original version of this Article contained errors in Equation 5.3, Equation 5.5, Equation 5.6 and Equation 5.13, where the lowercase characters were captured in a wrong font.

Equation 5.3

The $\varepsilon =0$ dynamics are decoupled, where the first oscillator, initialized at $({q}_{1}(0),{p}_{1}(0))=(q,p)$, follows a trajectory characterized by periodic clockwise circular rotation in phase space, while the second oscillator remains immobile:

$${q}_{1}(t)=qcost+psint, {p}_{1}(t)=pcost-qsint.$$

now reads:

The $\varepsilon =0$ dynamics are decoupled, where the first oscillator, initialized at $\left(q_1(0),p_1(0)\right)=({\mathcalligra{q}},{\mathcalligra{p}})$, follows a trajectory characterized by periodic clockwise circular rotation in phase space, while the second oscillator remains immobile:

$$q_1(t)=\mathcalligra{q} \cos{t}+\mathcalligra{p} \sin{t},\qquad\qquad p_1(t)=\mathcalligra{p} \cos{t} - \mathcalligra{q} \sin{t} .$$

Equation 5.5

$$=\frac{1}{2}({q}_{2}^{2}+{p}_{2}^{2})+\frac{{q}_{2}}{2\pi }{\int }_{0}^{2\pi }(qcost+psint)sin(2[qcost+psint]+2{q}_{2})dt$$

now reads:

$$=\frac{1}{2}(q_2^2+p_2^2)+\frac{q_2}{2\pi}\int_0^{2\pi}{ \left( \mathcalligra{q} \cos{t}+\mathcalligra{p} \sin{t} \right) \sin{\left(2\left[ \mathcalligra{q} \cos{t}+\mathcalligra{p} \sin{t}\right]+2q_2 \right)} dt}$$

Equation 5.6

$$=\frac{1}{2}({q}_{2}^{2}+{p}_{2}^{2})+{q}_{2}cos(2{q}_{2})\sqrt{{q}^{2}+{p}^{2}}{J}_{1}(\sqrt{{q}^{2}+{p}^{2}})$$

now reads:

$$=\frac{1}{2}(q_2^2+p_2^2)+q_2 \cos{(2 q_2)} \sqrt{ \mathcalligra{q}^2+\mathcalligra{p}^2} \ \mathcalligra{J}_1\left(2 \sqrt{ \mathcalligra{q}^2+\mathcalligra{p}^2} \right)$$

Equation 5.13

where in particular the (*q*, *p*) dynamics are now independent of $({Q}_{1},{Q}_{2},{P}_{1},{P}_{2})$ and can easily be solved for explicitly, given some initial conditions $(q(0),p(0))=(q,p)$:

$$q(t)=q+\varepsilon pt,p(t)=p.$$

now reads:

where in particular the (*q*, *p*) dynamics are now independent of $({Q}_{1},{Q}_{2},{P}_{1},{P}_{2})$ and can easily be solved for explicitly, given some initial conditions $\left(q(0),p(0)\right)=({\mathcalligra{q}}, {\mathcalligra{p}})$:

$$q(t)=\mathcalligra{q}+\epsilon \mathcalligra{p} t, \qquad p(t)=\mathcalligra{p}.$$

The original Article has been corrected.

